# Oxidative Stress Does Not Influence Subjective Pain Sensation in Inflammatory Bowel Disease Patients

**DOI:** 10.3390/antiox10081237

**Published:** 2021-08-01

**Authors:** Anna Krystyna Zielińska, Maciej Sałaga, Paweł Siwiński, Marcin Włodarczyk, Adam Dziki, Jakub Fichna

**Affiliations:** 1 Department of Biochemistry, Faculty of Medicine, Medical University of Lodz, Mazowiecka 6/8, 92-215 Lodz, Poland; ania.zielinska0122@gmail.com (A.K.Z.); maciej.salaga@umed.lodz.pl (M.S.); 2 Department of General and Colorectal Surgery, Faculty of Medicine, Medical University of Lodz, Żeromskiego 113, 90-549 Lodz, Poland; siwinski.pw@gmail.com (P.S.); dr.mwlodarczyk@gmail.com (M.W.); prof.dziki@gmail.com (A.D.)

**Keywords:** inflammatory bowel diseases, Crohn’s disease, ulcerative colitis, oxidative stress, pain

## Abstract

Oxidative stress (OS) has been proposed as a significant causative and propagating factor in inflammatory bowel diseases (IBDs). Modulation of OS is possible through antioxidants and inhibition of oxidizing enzymes. Thirty-one IBD patients and thirty-two controls were included in the study. The aim was to examine the levels of OS in colonic tissue of IBD requiring surgical intervention and control group, and their association with pain intensity. Total antioxidant capacity (TAC), superoxide dismutase (SOD) and catalase (CAT) activity, glutathione (GSH) and oxidized glutathione (GSSG) levels, and glutathione peroxidase (GPX) activity as markers of antioxidant defense were determined. Cyclooxygenases activities (Total COX, COX-1 and COX-2) were measured as prooxidant enzymes. Thiobarbituric acid reactive substances (TBARS) concentrations were measured to evaluate lipid peroxidation. Disease activity was assessed, and each subject filled out VAS and Laitinen’s pain assessment scales. Correlation between the OS, pain intensity, disease activity parameters, C-reactive protein (CRP), number of stools passed daily, disease duration, and dietary habits was investigated. No TAC differences were found between the groups. A significant decrease of SOD activity and GSH and GSSG levels was seen in IBD patients vs. controls, while GPX activity was diminished significantly only in CD patients. CAT and COX-1 activity was increased, and COX-2 significantly decreased in IBD. TBARS were significantly higher in CD patients compared to control group. No correlation was found between pain scores, inflammatory status, disease activity, disease duration, or dietary habits and OS markers. In our study, OS did not influence pain sensation reported by IBD patients.

## 1. Introduction

Inflammatory bowel diseases (IBDs), including Crohn’s disease (CD) and ulcerative colitis (UC), are chronic ailments of multifactorial etiology, affecting mostly the gastrointestinal (GI) tract [1]. IBD is a result of a complex interplay between genetic factors, changes in the colonic barrier function, immune system, intestinal microbiota, and environmental factors, such as diet, smoking, drugs, alcohol, or xenobiotics [1,2]. Constant exposure of the GI tract to risk factors leads to oxidative stress (OS): an overproduction of reactive oxygen species (ROS) and free radicals with accompanying insufficiency of endogenous antioxidant systems [3]. OS leads to the damage of the GI’s mucosal layer, which in turn stimulates immune response [4]. The imbalance between antioxidant systems and overproduction of ROS, generated mostly by phagocytic immune cells that infiltrate the mucosal tissue [2,3,4], disrupts cellular homeostasis, as it leads to cell malfunction and injury due to lipid, protein, carbohydrate and nucleic acid damage, thus accelerating and aggravating the inflammation [2,5]. OS, together with the following perpetuation of inflammatory reaction caused by overabundance of ROS, have been proposed as mechanisms involved in the onset, progression, and symptomatology of IBD. Antioxidant production is the first-line defense against ROS, however in a state of continuous OS those resources are diminished and IBD patients show reduced activity of main antioxidant systems and decreased detoxification [2,5].

IBD symptoms are diverse and non-specific. General manifestations such as weakness and weight loss are accompanied by intestinal granulomas, obstructions, strictures, fistulas, and abscesses throughout the GI tract [6,7]. OS is considered to be involved in several signals and symptoms of IBD, including diarrhea and abdominal pain [8]. Chronic abdominal pain is a common problem: 70% IBD patients suffer from pain during the exacerbation, and more than 30% during clinical remission of the disease [7,9]. Moreover, pain severity does not always correspond with clinical or endoscopic activity of the disease: 20 to 50% of IBD sufferers report continuation of chronic pain during periods of remission [7]. Pain is a symptom dramatically diminishing the quality of life, especially that a relapsing course of the disease causes intermittent exacerbations of pain [8,10]. Pain in IBD is also influenced by frequently reported fatigue, psychological factors, or prior surgeries [9]. Moreover, accompanying irritable bowel syndrome, visceral hypersensitivity, changes in sensory pathways during active inflammation, or bacterial overgrowth can lead to pain in IBD [9,11]. Reduction of abdominal pain is one of the main targets in IBD patients, who strive to lead an independent, symptom-free life [7]. However, pharmacological treatment of IBD, aiming to minimize the symptoms caused by functional and structural abnormalities, is often a cause of adverse effects. Corticosteroids and immunosuppressive agents can cause or further enhance diarrhea, cramping, as well as abdominal pain [8]. Opioids cause psychological and disease-related risks and their prolonged use (reported in 3 to 30% of IBD patients) may cause neuroplastic changes that result in bowel hyperalgesia and intensification of pain [7]. Modulation of OS by enhancing antioxidant systems or inhibiting oxidizing enzymes could positively influence the course of the disease. The antioxidant status can be estimated by measurements of total antioxidant capacity (TAC), which is a sum of food-derived and endogenous antioxidants. Superoxide dismutase (SOD) and catalase (CAT) are important endogenous antioxidant enzymes. Glutathione (GSH) is a nonenzymatic molecule which protects cells against the overproduction of free radicals. Glutathione peroxidase (GPX), produced mainly by intestinal epithelium, regulates the amount of GSH by catalyzing it into oxidized glutathione (GSSG) [2,3].

On the other hand, multiple enzymes, such as cyclooxygenases (COX) participate in endogenous production of ROS; in line, COX-1 and COX-2 are up-regulated in human ulcers, while COX-2 is strictly connected to precancerous changes in inflamed colonic mucosa [4]. Lipid peroxidation, the oxidative damage of membrane lipids, unless disrupted by antioxidants at initiation, spreads quickly in a self-perpetuating, unstoppable chain reaction that intensifies the oxidative destruction [5,6]. The marker of this process is Thiobarbituric Acid Reactive Substances (TBARS) level [12]. Estimation of TBARS reflects the malondialdehyde (MDA) concentration- the final product of lipid peroxidation [13].

The relation between OS status and nociception itself has been evident for decades, although it has never been investigated in the context of colonic tissue in IBD. We aimed at determining if there is an association between the above- mentioned OS markers measured in the colonic mucosa and the severity of pain experienced by IBD patients who suffered disease complications unresponsive to pharmacological treatment and needing surgery. Moreover, we took a closer look at the possible correlation of anti- and prooxidants with the disease activity and the severity of inflammation measured by C-reactive protein (CRP). We also wanted to find out how the markers of OS differ in CD and UC compared to controls.

## 2. Materials and Methods

### 2.1. Ethical Considerations

The study was conducted in accordance with the ethical principles of the 1975 Declaration of Helsinki and the study protocol was approved by the Committee of Bioethics of Medical University of Lodz, Poland (RNN/305/18/KE, 16 October 2018).

### 2.2. Patients

This prospective observational study was conducted in 31 IBD patients and 32 controls of Caucasian origin; 47.6% were male. Median age was 50.5 years (range 20 to 91 yrs). Subjects from the study group were IBD patients who underwent elective or emergent surgical treatment due to the disease complications or exacerbation not reacting to pharmacological treatment in the Department of General and Colorectal Surgery, Medical University of Lodz, Poland. Only adult patients with IBD were enrolled (CD nL’18 and UC nL’13).

**Figure 1:**
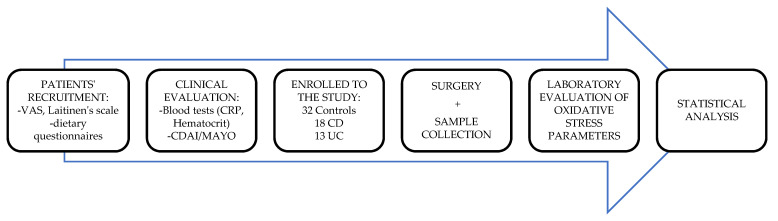
Study design.

Control group consisted of patients suffering from various non-inflammatory colon entities (Table 1), undergoing elective surgical resection of the involved bowel part, in the same department. The group was homogenous to the study group in terms of sex and body mass index. Study design is presented in Figure 1.

### 2.3. Inclusion Criteria

The inclusion criteria for the study groups were based on the proper diagnosis according to clinical, radiological, endoscopic and histological criteria recommended by the European Crohn’s and Colitis Organization (ECCO).

Patients with a history of cardiovascular disease, pulmonary and kidney disease, allergy, diabetes, lichen planus, psoriasis, atopic dermatitis and other autoimmune skin lesions and those treated with anti-inflammatory drugs, antioxidants or statins were excluded from the study.

### 2.4. Material Collection

After the participants from both groups gave a written consent, about 5 cm wide fragments of resected bowel were collected intraoperatively. The samples were collected into plastic containers for sample storing and immediately frozen at −80 °C and stored until use. Before assaying, bowel samples were divided in 50 milligram pieces, carefully weighed and moved to 2-milliliter capped eppendorf tubes.

Additionally, the day prior to the operation blood samples were obtained for standard blood work and clinical evaluation.

### 2.5. Determination of the Antioxidant Capacity

Total antioxidant capacity (TAC) was measured using Antioxidant Assay Kit (Cayman Chemicals, Ann Arbor, MI, USA), which relies on the ability of antioxidants in the sample to inhibit the oxidation of ABTS^®^ (2,2′-azino-di-[3-ethylbenzthiazoline sulphonate]) to ABTS^®^ + by metmyoglobin. Cell lysate was prepared by centrifugation (2000× *g* for 10 min at 4 °C) and cell pellet was homogenized on ice in cold 5 mM potassium phosphate, pH = 7.4 containing 0.9% sodium chloride and 0.1% glucose per gram of tissue. Centrifugation of homogenates at 10,000× *g* for 15 min at 4 °C was performed. Collected supernatants were assayed following the assay protocol. Then the absorbance was recorded at 750 nm in a microplate reader (iMARK MicroPlate Reader; Bio-Rad, Hercules, CA, USA). All measurements were performed in duplicate. Antioxidant concentration was presented in millimoles per milligram of wet tissue. Antioxidant concentration was calculated from a standard curve using lyophilized powder of Trolox (6-hydroxy-2,5,7,8-tetramethylchroman-2-carboxylic acid) reconstituted in 1 mL of HPLC-grade water.

### 2.6. Determination of SOD Activity

SOD activity in the colonic tissue was established with help of Superoxide Dismutase Assay Kit (Cayman Chemicals, Ann Arbor, MI, USA), that uses tetrazolium salt for detection of superoxide radical, which is produced by xanthine oxidase and hypoxanthine. Tissue samples were undergoing homogenization in ice-cold 20 mM HEPES buffer, pH = 7.2, containing 1 mM ethylene glycol-bis(2-aminoethylether)-N,N,N′,N′-tetra acetic acid (EGTA), 210 mM mannitol and 70 mM sucrose per gram of tissue. Next, Centrifugation of homogenates at 1500× *g* for 5 min at 4 °C was performed. Collected supernatants were assayed following the assay protocol. Absorbance was measured at 450 nm (iMARK Microplate Reader, Bio-Rad, Hercules, CA, USA). All measurements were performed in duplicate. SOD activity was demonstrated in units per milligram of wet tissue (1 U equals the quantity of enzyme needed for 50% dismutation of the superoxide radical). Units of SOD activity were calculated from a standard curve using purified bovine erythrocyte SOD enzyme.

### 2.7. Determination of CAT Activity

CAT activity in the colonic tissue was established with help of Catalase Assay Kit (Cayman Chemicals, Ann Arbor, MI, USA), using the peroxidative function of CAT for determination of its enzymatic activity. This method uses the reaction of CAT with methanol in the presence of hydrogen peroxide. Tissue samples underwent homogenization in the ice-cold buffer that contains 50 mM potassium phosphate, pH = 7.0 and 1 mM EDTA per gram of tissue. Centrifugation of homogenates at 10,000× *g* for 15 min at 4 °C was performed Collected supernatants (put on ice) were assayed following the assay protocol. The samples were diluted in a ratio of 1:9 with Catalase Sample Buffer included in the assay. Absorbance was measured at 540 nm (iMARK Microplate Reader, Biorad, UK). All measurements were performed in duplicate. CAT activity was demonstrated in U per milligram of wet tissue (1 U equals the amount of enzyme causing the formation of 1.0 nM of formaldehyde per minute at 25 °C). Units of CAT activity were calculated from standard curve using purified bovine liver CAT enzyme.

### 2.8. Measurement of Total GSH and GSSG Levels

Quantities of Total GSH and GSSG in the colonic tissue were established with the help of a Glutathione Assay Kit (Cayman Chemicals, Ann Arbor, MI, USA), using an enzymatic recycling method that utilizes glutathione reductase, for the measurement of total GSH. The sulfhydryl group of GSH reacts with DTNB (5,5′-dithio-bis-2-(nitrobenzoic acid), Ellman’s reagent) and produces yellow colored 5-thio-2-nitrobenzoic acid (TNB). The mixed disulfide, GSTNB (between GSH and TNB) that is synchronously produced, is reduced by GR to recycle the GSH and produce more TNB. The speed of TNB formulation is directly proportional to this recycling reaction. This reaction in turn is directly proportional to the concentration of GSH in the sample. GSH is easily oxidized to the disulfide dimer GSSG, produced during the reduction of hydroperoxides by glutathione peroxidase. GSSG is reduced to GSH by glutathione reductase. Quantification of GSSG, exclusive of GSH, is accomplished by first derivatizing GSH with 2-vinylpyridine. Tissue samples underwent homogenization in cold 50mM 2-(N-morpholino)-ethanesulfonic acid (MES) buffer, pH = 7.0 that contains 1 mM EDTA per gram of tissue. Centrifugation of homogenates at 10,000× *g* for 15 min at 4 °C was subsequently performed. Collected supernatants (put on ice) were assayed following the assay protocol. Absorbance was measured at 410 nm (iMARK Microplate Reader, Bio-Rad, UK) after 25 min. All measurements were performed in duplicate. Total GSH and GSSG concentrations were read off a standard curve and expressed as µM of GSH and GSSG per milligram of wet tissue.

### 2.9. Determination of GPX Activity

GPX activity in the colonic tissue was established with help of Glutathione Peroxidase Assay Kit (Cayman Chemicals, Ann Arbor, MI, USA), assessing indirectly GPX activity by a coupled reaction with glutathione reductase (GR). Oxidized glutathione–GSSG that is generated upon reduction of hydroperoxide by GPX, is recycled to its reduced state by GR and NADPH. The oxidation of NADPH to NADP+ is followed by a decrease in absorbance at 340 nm. Tissue samples underwent homogenization in cold 50 mM Tris-HCl buffer, pH = 7.5 that contains 5 mM EDTA, and 1 mM Dithiothreitol (DTT) per gram of tissue. Centrifugation of homogenates at 10,000× *g* for 15 min at 4 °C was subsequently performed. Collected supernatants (put on ice) were assayed following the assay protocol. Absorbance was measured at 340 nm (iMARK Microplate Reader, Bio-Rad, UK). All measurements were performed in duplicate. GPX activity was demonstrated in units per milligram of wet tissue (1 U equals the amount of enzyme that will cause the oxidation of 1.0 nmol of NADPH to NADP+ per minute at 25 °C).

### 2.10. Determination of Total COX, COX-1 and COX-2 Activity

Total COX, COX-1, and COX-2 activity in the colonic tissue were established with help of COX Fluorescent Activity Assay Kit (Cayman Chemicals, Ann Arbor, MI, USA), which utilizes the peroxidase component of COXs. The reaction between hydroperoxy endoperoxide (PGG_2_) and ADPH (10-acetyl-3,70 dihydroxyphenoxazine) produces highly fluorescent compound: resorufin. Its fluorescence can be easily analyzed with an excitation wavelength between 530–540 nm and an emission wavelength between 585–595 nm. The kit included isozyme-specific inhibitors for distinguishing COX-2 activity from COX-1 activity. Tissue samples were homogenized in cold 50 mM Tris-HCl buffer, pH = 7.5 containing protease inhibitor- Pierce™ Protease and Phosphatase Inhibitor (ThermoScientific, Waltham, MA, USA). Centrifugation of homogenates at 10,000× *g* for 15 min at 4 °C was subsequently performed. Collected supernatants (put on ice) were assayed following the assay protocol. Fluorescence was measured with an excitation wavelength of 530 nm and an emission wavelength of 590 nm (Victor X Microplate Reader, PerkinElmer, USA). All measurements were performed in duplicate. Total COX activity was demonstrated in units per milligram of wet tissue (1 U equals the amount of enzyme that will cause the formation of 1 nmol of fluorophore per minute at 22 °C). Fluorophore concentrations of the samples were calculated from standard curve using resorufin standard.

For measurements of COX-1 and COX-2, DuP-697- a potent and time-dependent inhibitor of COX-2 was used. The assay followed the same procedure as described above. Total COX Activities of DuP- 697 treated samples were subtracted from the Total COX Activity of their corresponding samples and then divided by the Total COX Activity of the samples and multiplied by 100 to give the percent inhibition. The amount of inhibition corresponded to the amount of COX-2 in the sample. Through subtracting the COX-2 from Total COX we calculated the amount of COX-1, and then presented them as percentage.

### 2.11. Measurement of TBARS

Thiobarbituric Acid Reactive Substances (TBARS) were measured using TBARS Assay Kit (Cayman Chemicals, Ann Arbor, MI, USA), which is a well-established method for screening and monitoring lipid peroxidation and relies on the measurement of MDA-TBA adduct formed by the reaction of MDA (malondialdehyde)—a naturally occurring product of lipid peroxidation, and TBA (thiobarbituric acid) under high temperature (100 °C) and acidic conditions. Tissue samples were homogenized on ice in cold Radio-Immunoprecipitation Assay (RIPA) buffer containing 1 mM EDTA per gram of tissue. Centrifugation of homogenates at 1600× *g* for 15 min at 4 °C was subsequently performed. Collected supernatants (put on ice) were assayed following the assay protocol. Then the absorbance was recorded at 540 nm in a microplate reader (iMARK MicroPlate Reader; Bio-Rad, USA). All measurements were performed in duplicate. MDA concentration was expressed in micromoles per milligram of wet tissue. MDA concentration was calculated from a standard curve using TBA Malondialdehyde Standard.

### 2.12. Demographic and Clinical Data

Before the surgeries, each subject filled out dietary, vitamin D supplementation, and pain questionnaires (described below). Moreover, IBD patients were asked questions concerning the duration of the disease, extraintestinal symptoms and general wellbeing. The demographic data including age, gender, body mass index, duration of the disease, patient’s age at diagnosis and previous biological treatment, data concerning alcohol, and drug consumption and smoking history were collected from the patients and analyzed.

Laboratory evaluation of all patients included measurements of C-reactive protein (CRP), determined using automatic devices. Measurements were performed one day prior to the operation (Table 2).

### 2.13. Assessment of Disease Activity

The disease activities of enrolled patients were assessed by using validated scales including Crohn’s Disease Activity Index (CDAI) for CD and endoscopic Mayo Score for UC, respectively as well as CRP levels and number of stools passed daily prior to the surgery [14]. According to ECCO, CDAI < 150 was defined as remission; CDAI 150–220 with no features of obstruction, fever, dehydration, abdominal mass or tenderness was defined as mild CD; CDAI 220–450 or intermittent vomiting, or weight loss >10% or treatment for mild disease ineffective, or tender mass with no overt obstruction was defined as moderate CD; CDAI > 450 or cachexia (body mass index (BMI) < 18 kg/m^2^), or evidence of obstruction or abscess or persistent symptoms despite intensive treatment was defined as severe CD.

For UC, clinical remission was defined as a Mayo Score of 0; mild UC was defined as Mayo Score of 1; moderate UC was defined as Mayo Score of 2; severe UC was defined as Mayo Score of 3.

### 2.14. Assessment of Pain Experienced by the Patient

Before the surgery, all the patients were asked to fill out two pain questionnaires: visual analog scale (VAS), which is a validated, subjective measure for acute and chronic pain [15]. Scores are recorded by making a handwritten mark on a 10-cm line that represents a continuum between “no pain”—0 and “worst pain”—10 and Laitinen Pain Scale, which allows measuring pain intensity while simultaneously assessing other factors accompanying pain, namely: frequency of pain occurrence, taking pain killers and limiting motor activity [16]. The scale and mean results of the questionnaire are presented in Table 3. For simplification of the statistical analysis, Laitinen’s scale points have been added.

### 2.15. Statistical Analysis

The data were analyzed using the GraphPad Prism 8.0.1. (GraphPad Software, United States). Continuous demographic and biochemical data are presented as means ± standard deviation (SD), demographic categorical data were described with absolute frequencies and percentages. An analysis of variance (One-way ANOVA with repeated measures) was used to detect differences. Dunnett test was used to correct for multiple comparisons. Shapiro–Wilk’s W test was used to test the normality of distribution of the variables. Multiple linear regression was applied to assess the correlations. Pearson’s test was used for continuous variables or Spearman’s test was used for categorical variables were used to calculate *p* values. A value of *p* < 0.05 was considered statistically significant.

## 3. Results

### 3.1. Study Group

The demographics, laboratory outcomes and disease characteristics of the study population are summarized in Table 1; Table 2.

A total of 31 IBD patients qualified for surgical treatment (18 CD and 13 UC) and 32 controls were enrolled to the study. In IBD group the majority were female, with no significant difference in age between CD and UC patients. The questionnaires and medical charts of included IBD subjects were reviewed and Total antioxidant capacity (TAC), SOD activity, CAT activity, total GSH and GSSG levels, GPX activity, Total COX, COX-1, and COX-2 activity and TBARS levels were measured in the bowel tissue and then compared with control group’s results.

### 3.2. Antioxidant Systems

**Figure 2:**
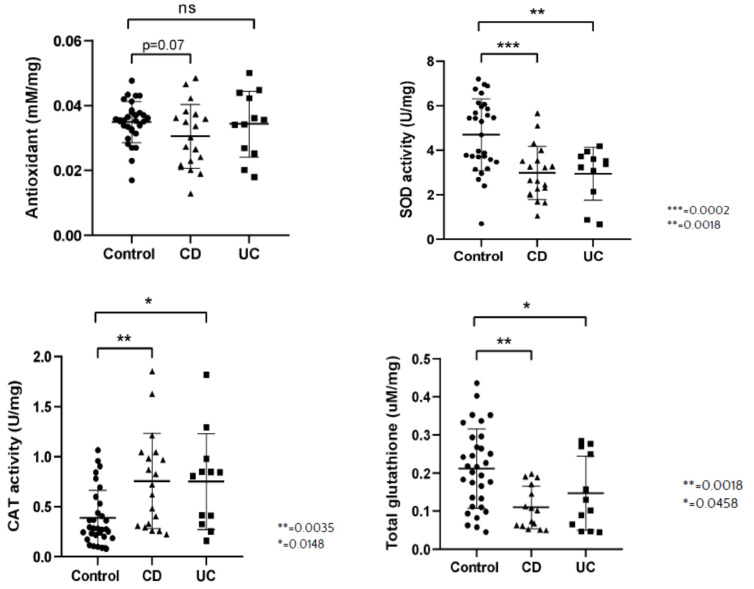

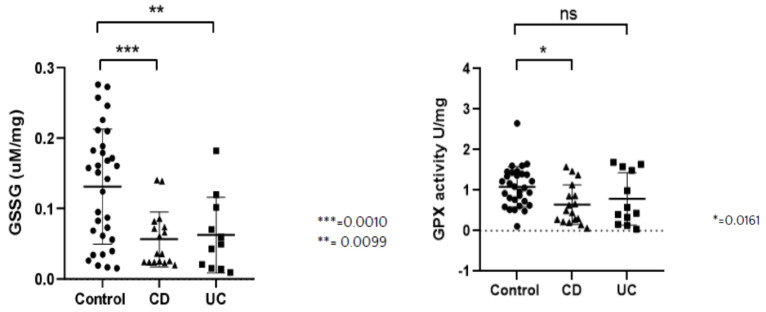
Total antioxidant concentration (TAC) (mM/mg sample), activity of superoxide dismutase (SOD, U/mg sample), activity of catalase (CAT, U/mg sample), concentration of total glutathione (GSH, uM/mg sample), concentration of oxidized glutathione (GSSG, uM/mg sample) and activity of glutathione peroxidase (GPX, U/mg sample) per milligram tissue sample in healthy subjects (Control), in patients with Crohn’s disease (CD) and ulcerative colitis (UC).

We discovered that tissue TAC was not significantly different in all groups, however *p* = 0.07 for the control vs. CD group was obtained. On the other hand, SOD activity was significantly increased in control group compared to CD and UC. CAT activity was lower in control group in comparison to CD and UC. Total GSH and GSSG levels were significantly higher in control group in comparison to CD and UC patients. GPX activity was significantly higher in control group compared to CD, but it lacked statistical significance compared with UC (Figure 2).

### 3.3. Oxidative Stress

**Figure 3:**
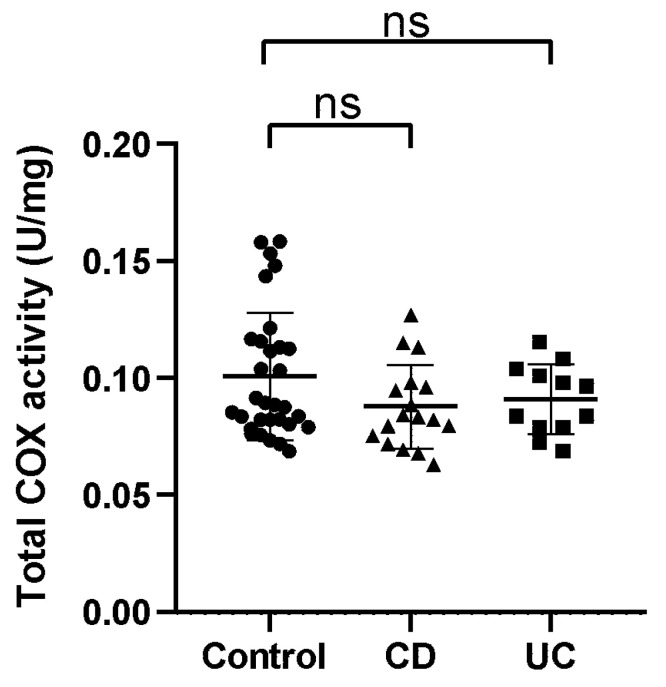

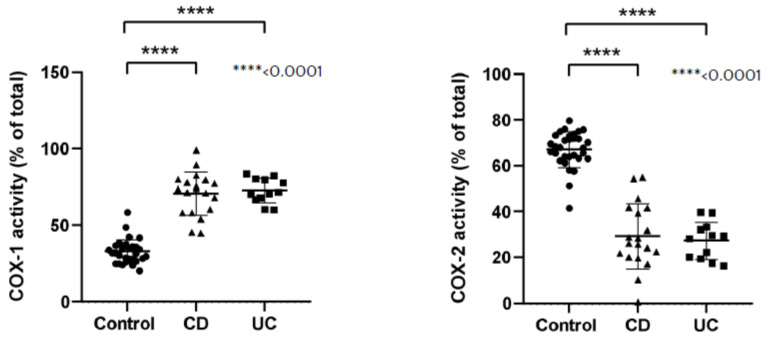
Activity of total cyclooxygenase (COX, U/mg sample), cyclooxygenase-1 isoform (COX-1, percentage of total COX activity) and cyclooxygenase-2 isoform (COX-2, percentage of total COX activity) per milligram tissue sample in healthy subjects (Control), in patients with Crohn’s disease (CD) and ulcerative colitis (UC).

While measuring the total COX activity in the colonic tissues, we found no significant differences between the groups. COX-1 activity was significantly higher in CD and UC groups, while COX-2 activity was significantly lower in those groups in comparison to the control group (Figure 3).

### 3.4. Lipid Peroxidation

**Figure 4:**
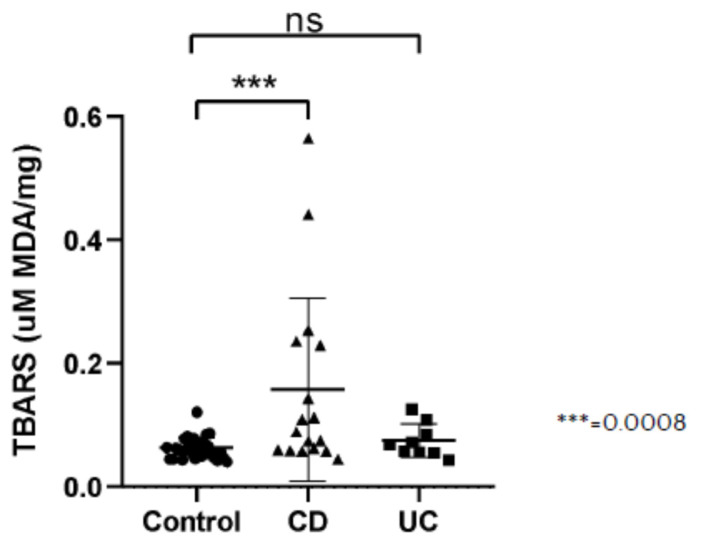
Thiobarbituric Acid Reactive Substances (TBARS) presented as concentration of malondialdehyde (MDA) per milligram tissue sample (uM MDA/mg sample) in healthy subjects (Control), in patients with Crohn’s disease (CD) and ulcerative colitis (UC).

TBARS measured as concentration of MDA were significantly higher in the CD group in comparison to controls. The statistically significant difference was not present between the UC and control group (Figure 4).

### 3.5. Disease Activity

Mean CRP in IBD patients in our study equaled 66.04 ± 64.27. The CRP levels did not correlate with investigated markers (TAC, SOD activity, CAT activity, total GSH and GSSG levels, GPX activity, Total COX, COX-1 and COX-2 activity, and TBARS levels.)

The majority of CD patients presented with a moderately active disease, with mean CDAI of 305.61 ± 109.87. In UC, the course of the disease was severe and most of the UC patients were scored with 3 in endoscopic Mayo assessment. Mean endoscopic Mayo Score was 2.65 ± 0.74.

**Figure 5:**
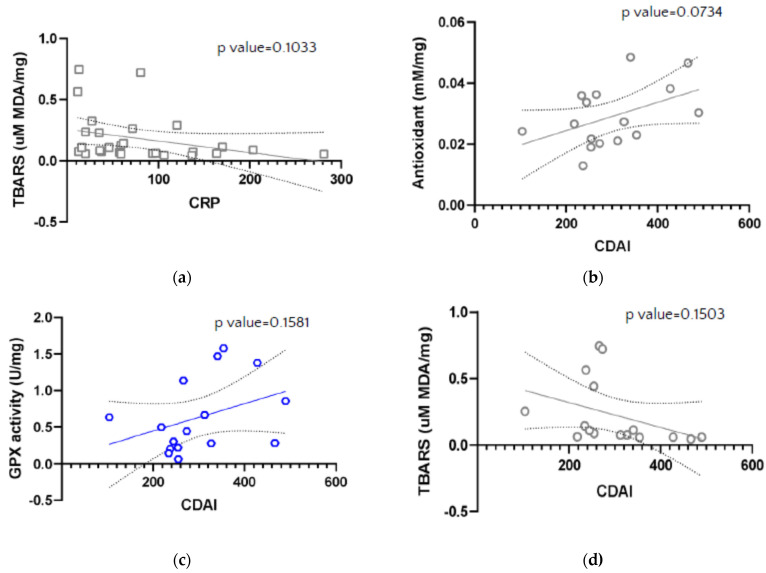
Correlation between Thiobarbituric Acid Reactive Substances (TBARS) presented as concentration of malondialdehyde (MDA, uM MDA/mg sample) and value of C-reactive protein (CRP) (**a**), and between antioxidant concentration (mM/mg) (**b**), GPX activity (U/mg) (**c**), Thiobarbituric Acid Reactive Substances (TBARS) presented as concentration of malondialdehyde (MDA, uM MDA/mg sample) (**d**) and Crohn’s Disease Activity Index (CDAI) in IBD patients.

CDAI and Mayo scores were analyzed for association with colonic tissue levels of TAC, SOD activity, CAT activity, total GSH and GSSG levels, GPX activity, Total COX, COX-1 and COX-2 activity, and TBARS levels; however, no significant correlations were found (Figure 5).

### 3.6. Pain and Other Disease Symptoms

Pain and other individual variables were analyzed for correlations with colonic tissue TAC, SOD activity, CAT activity, total GSH and GSSG levels, GPX activity, Total COX, COX-1 and COX-2 activity, and TBARS levels, as described in the methods section.

**Figure 6:**
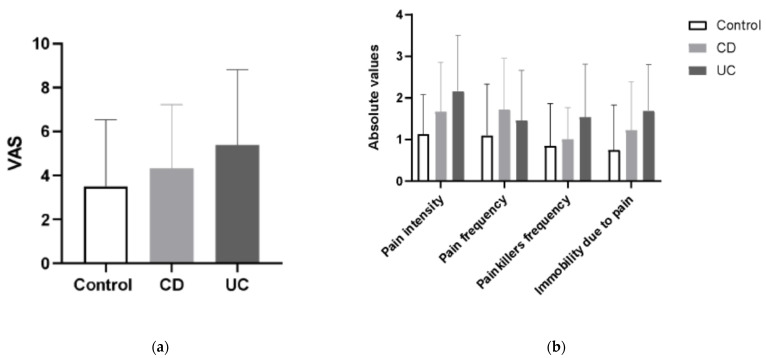
The mean values of visual analog scale (VAS) for quantification of disease-related pain (**a**) and the differences in the values of modified Laitinen Pain Scale (**b**) in controls, CD and UC patients.

Pain experienced and subjectively assessed by IBD and control patients in VAS Pain Scale and Laitinen’s Scale was greater in CD and UC patients than in controls, with UC patients presenting with more severe pain in comparison to CD. Those differences however did not reach statistical significance (Figure 6). Presence of pain was reported by 100% of IBD of patients, and by 78.12% patients in the control group (25/32).

**Figure 7:**
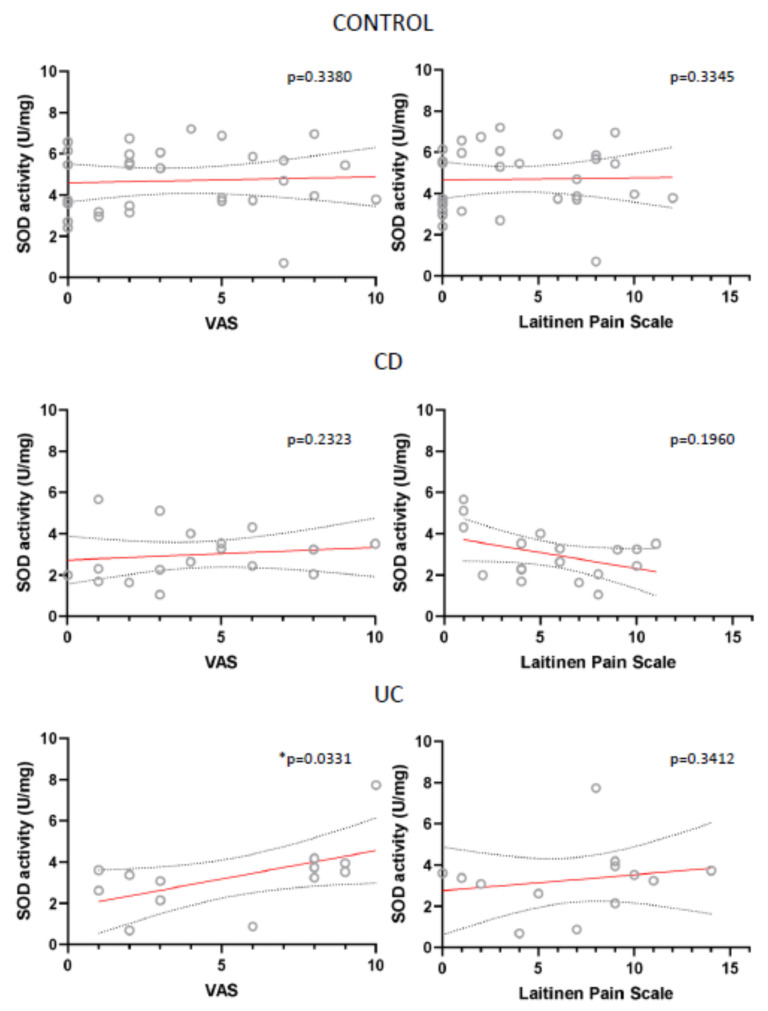
Correlation between the mean values of visual analog scale (VAS) for quantification of disease-related pain, the values of modified Laitinen Pain Scale and SOD activity (U/mg sample) in controls, CD and UC patients.

**Figure 8:**
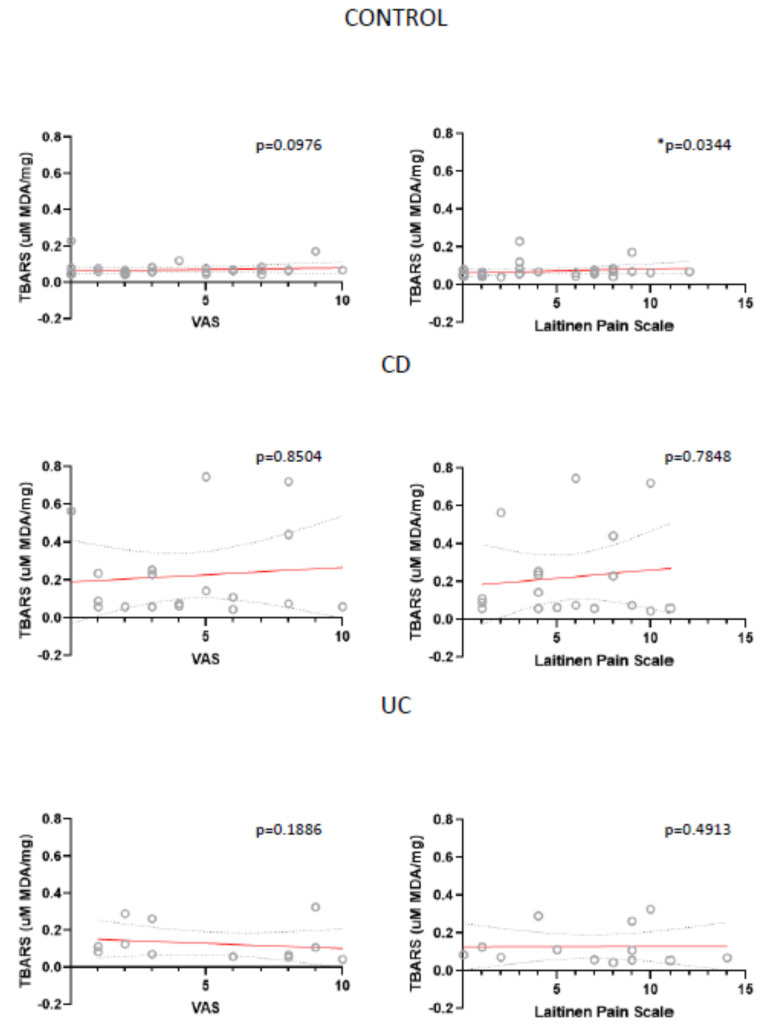
Correlation between the mean values of visual analog scale (VAS) for quantification of disease-related pain, the values of modified Laitinen Pain Scale and Thiobarbituric Acid Reactive Substances (TBARS) presented as concentration of malondialdehyde (uM MDA/mg sample) in controls, CD and UC patients.

Also, for all analysed parameters we only found two statistically important correlations: between VAS and SOD in UC patients and between Laitinen’s Scale and TBARS in control patients (Figure 7; Figure 8).

**Figure 9:**
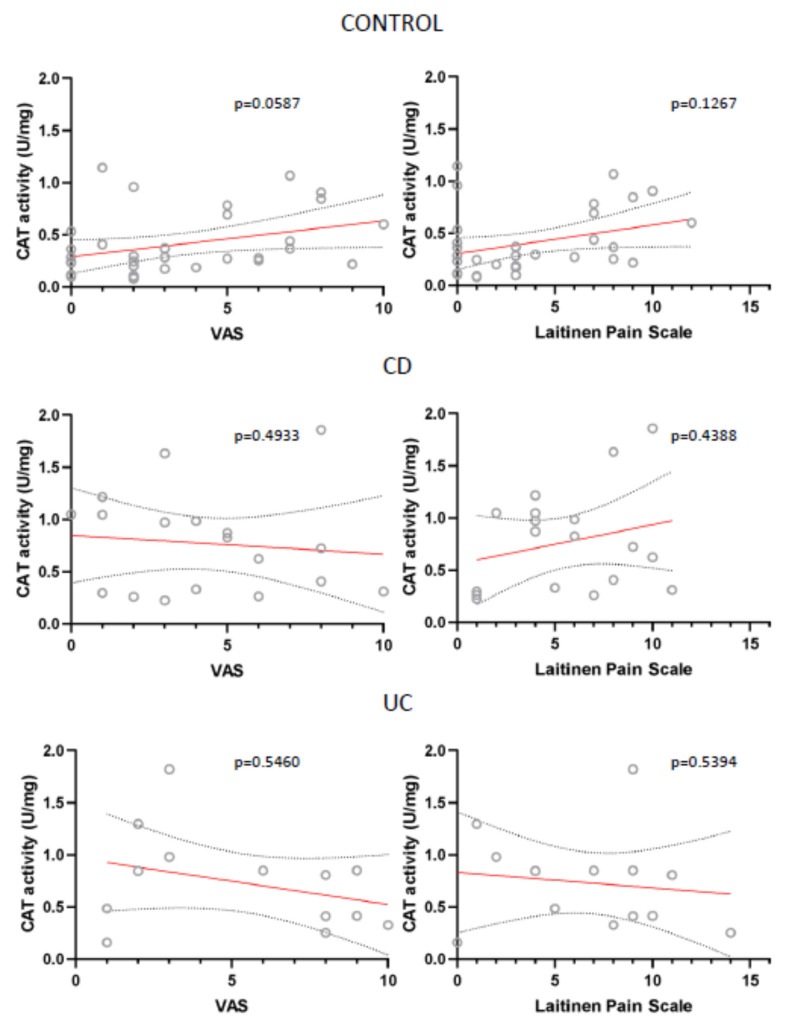
Correlation between the mean values of visual analog scale (VAS) for quantification of disease-related pain, the values of modified Laitinen Pain Scale and CAT activity (U/mg sample) in controls, CD and UC patients.

**Figure 10:**
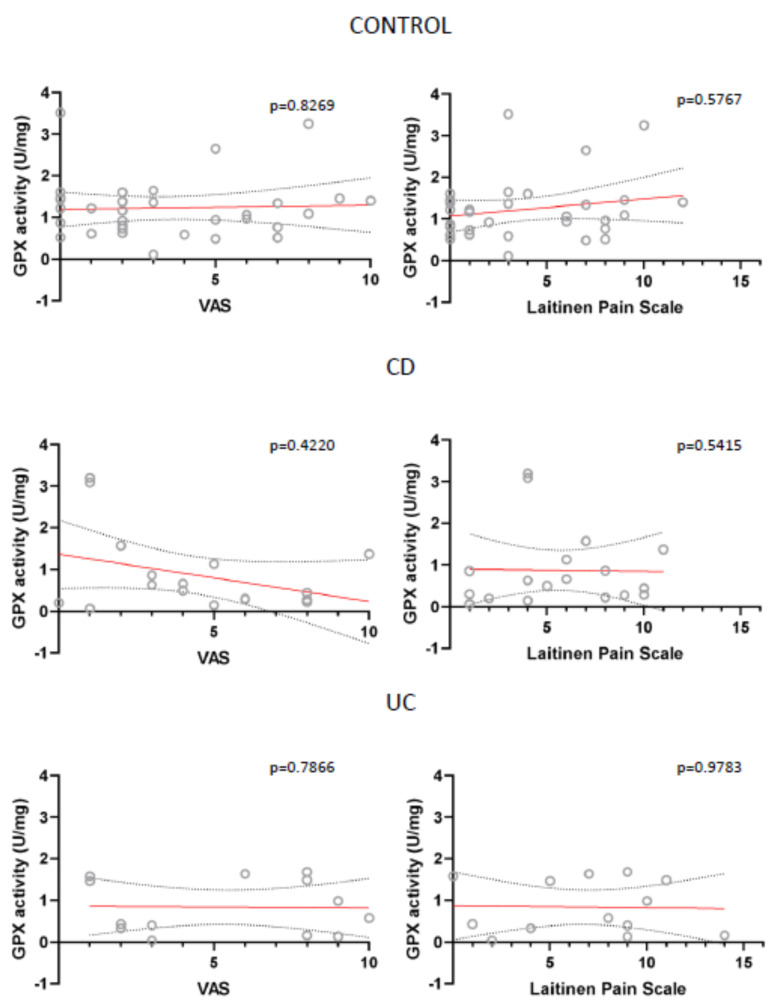
Correlation between the mean values of visual analog scale (VAS) for quantification of disease-related pain, the values of modified Laitinen Pain Scale and GPX activity (U/mg sample) in controls, CD and UC patients.

Other parameters, like CAT and GPX activity were not statistically important, have however been close to reaching it (Figure 9; Figure 10). This correlation was also absent in case of TAC, total GSH and GSSG levels, Total COX, COX-1 and COX-2 activity (data not shown).

No correlation was found between the OS markers and the extraintestinal symptoms, duration of the disease, use of painkillers, defecation and body mass index. Dietary habits of IBD patients and vitamin D supplementation also did not influence the antioxidant defense and prooxidant activity in the colonic tissue, therefore those variables were not included.

## 4. Discussion

We found changes in the antioxidant profile in IBD patients unresponsive to pharmacological treatment when compared with controls: SOD activity, and total GSH and GSSG levels were decreased in both CD and UC patients, whereas GPX activity significantly decreased only in case of CD vs. controls. TAC did not differ significantly between IBD patients and healthy controls; however, the CD group almost reached statistical importance, with *p* = 0.07. Furthermore, CAT activity was elevated in CD and UC patients in comparison to control group. No changes in total COX activity were found between the groups, whereas COX-1 activity was elevated in IBD. OS estimated by measuring lipid peroxidation products TBARS was significantly elevated in CD; however, there was no difference between UC and controls. OS markers in CD, UC, and control group were analyzed in connection to pain intensity. We did not find any association between OS markers and pain severity or frequency. Moreover, no associations were found with disease activity and inflammatory status. Dietary habits and vitamin D supplementation did not add any statistically significant information to the study.

TAC or total antioxidant status (TAS) is a cumulative measure that reflects power to resist the prooxidant action, to which all the present antioxidants are contributing [5]. Our study did not find any statistically important differences between CD, UC and control group. In most of the studies performed on adults, TAC/TAS measured in blood serum or plasma are decreased in IBD patients, both those suffering from CD and UC [17]. For example, Yuksel at al. measured TAS, total oxidant status (TOS) and oxidative stress index (OSI) in the serum of 80 IBD patients and 80 controls. TAS level was lower, whereas TOS and OSI higher in IBD compared to control group [18]. However, in a study by Luceri et al. no difference in TAS measured in the serum was observed in CD patients compared to controls, despite the high degree of OS observed in CD [19]. In contrary, Pereira et al. described increased TAS in IBD [20]. Sampietro et al. stated that the imbalance between pro- and antioxidant mechanisms in CD can be reduced by a conservative surgery, as after the resection TAS levels were comparable to those in healthy persons [21]. When measured in saliva, TAS levels were decreased in CD patients according to studies performed by Rezaie et al. and Jahanshahi et al. [22,23]. In a study by Szczeklik et al., in which both serum and saliva TAC were measured, serum TAC was lower in active CD in comparison to inactive CD and healthy controls. Saliva TAC did not differ significantly between active and inactive CD; however, they were decreased in comparison to healthy controls [24].

To preserve cellular homeostasis, the activity of prooxidants has to be compensated for by antioxidants. Antioxidant enzymes SOD, CAT, and GPX, are present in cells and have a primary role in detoxification [2]. Mrowicka et al. found significantly lower SOD, CAT and GPX levels in IBD patients in relation to controls [1]. Similarly, lower SOD activity was described by Alzoghaibi et al. [25], which is in accordance to our study. In a study by Achitei et al. serum SOD and GPX were reduced in CD patients with inactive disease but increased in active CD patients compared to controls, whereas Szczeklik et al. observed SOD down-regulation only in patients with active CD alternation [26,27]. Another study by Szczeklik et al. described reduced GPX and CAT activities in serum and saliva, confirming the previous results [24]. In our case, SOD and GPX were reduced in CD and UC compared to controls, but disease activity did not influence this outcome. Kruidenier at al. studied inflamed mucosa in CD patients and reported increased GPX activity in comparison to controls [28]. Contrarily, Pinto et al. presented reduced GPX activity in the inflamed CD mucosa compared to non-inflamed CD or healthy mucosa [29].

In our study CAT activity in CD and UC was increased in comparison to control group. Similar outcome was presented by Rana et al. in UC patients. It was however accompanied by increase in SOD and GPX as well, while total GSH level was significantly decreased compared to controls [30]. Krzystek- Korpacka et al. described diminished GPX and CAT activities in erythrocytes of IBD patients, with GPX being IBD’s indicative with 73% accuracy and CAT with 63% [31]. CAT activity measured in tissue homogenates was not significantly different between CD, UC, and control groups in a study by Bouzid et al. [32]. Sheethal et al. performed a study on mice with acetic acid-induced model of UC [13]. OS developed due to inflammatory condition led to amplified ROS generation in the colonic tissue with significant reduction of SOD, GPX, and CAT due to inefficacious scavenging of free radicals which increases ROS generation. This effect due to ulcer condition was considerably changed and antioxidant status improved after administration of antioxidant formula with powerful scavenging activity.

The second line of antioxidant defense consists of free radical scavengers neutralizing free radicals by donating electrons, including GSH. Our study showed an increase in colonic total GSH and GSSG. Sido et al. also stated that GSH redox status is diminished in inflamed ileum mucosa compared to non-inflamed mucosa in CD patients [33]. That was caused by greater OS in inflamed areas presenting as an increase of GSSG. Iantomasi et al. also stated that in diseased ileum of CD patients GSSG levels are higher than in the healthy tissue [34]. Studies by Dudzińska et al. showed that levels of GSH and GSSG in IBD patients do not show significant differences when compared to healthy controls, as well as CAT and SOD [6]. GSH was also found to be decreased in serum of patients with active CD or patients with complications [5]. Rana et al. found a decrease in GSH in erythrocytes and Homouda in serum of UC patients [30,35]. Serum and saliva GSH levels were also found by Abbas et al. to be significantly decreased [36]. In experimental colitis models, induced by dextran sulfate sodium (DSS), GSH levels are decreased and can be restored to normal levels by antioxidants [4]. Also, in the acetic acid-induced colitis model, treatment with antioxidant formula increased significantly the levels of GSH, which clearly indicates the preventive action against free radicals [13].

Multiple enzymes partake in endogenous ROS production by catalyzing chemical reactions, including COXs. We found no differences in Total COX activity in IBD patients, COX-1 however was significantly increased in IBD patients in comparison to healthy controls. COX-1 and COX-2 are generally up-regulated in ulcerous conditions [2]. Prooxidant enzymes, such as COX-2, have been found to be elevated in active UC and CD mucosa [37]. In acetic acid induced colitis, total COX and COX-2 activity was significantly increased, and antioxidant treatment normalized those levels. In colitis environment, when COX-2 increases, more prostaglandins are produced [13].

ROS have negative effects on mitochondrial and nuclear DNA, proteins and lipids. Lipid peroxidation alters transmembrane enzymes, transporters and receptors what leads to destruction of cell’s homeostasis and metabolism. Moreover, end products of lipid peroxidation cause protein damage [2]. In our study, lipid peroxidation measured by TBARS was elevated in CD, but not in UC or controls. Excess lipid peroxidation was presented as an important pathogenic factor in IBD by Pelli et al. [38]. Mrowicka at al. observed a decrease in MDA concentration in IBD patients, however the division between CD and UC patients was not reported [1]. Dudzińska et al. reported excess lipid peroxidation in CD patients, in both active and inactive disease [6]. Abbas et al. found increased MDA in serum and saliva of CD patients [36]. Luceri et al. also found elevated levels of serum TBARS in CD patients with severe relapse, suggesting that they could serve as prospective biomarkers for CD diagnosis and monitoring [19]. Boehm et al. described MDA to be a very good marker for CD, with 91% accuracy in discriminating CD patients from controls, which was confirmed by Szczeklik et al., who reported 87% accuracy [5,24,39].

Assessment of disease activity is crucial for appropriate therapeutic decisions. CRP is the most common marker of inflammation in IBD. In our study we failed at finding statistically important differences between CRP levels and OS markers. CDAI and Mayo Score were also not positively correlated with studied markers. Yuksel et al. in contrary found a positive correlation between the OS index and CRP in CD patients, which indicated a connection between excess OS and higher inflammation [18]. Moreover, treatment with antioxidants in acetic acid colitis models led to reduction in CRP levels [13]. Szczeklik et al. described a positive correlation between saliva MDA and GSH and CRP and CDAI [27]. In a study performed by Krzystek- Korpacka et al., GPX measured in erythrocytes was significantly diminished in active UD and CD patients and inversely related to CDAI and Mayo Score and had a comparable power to CRP in finding patients with active disease. Erythrocyte SOD activity was also positively correlated to CDAI in CD, but not to UC, and CAT activity was down- regulated more in active, than inactive CD [31].

To the best of our knowledge, this is the first study attempting to determine the connection between overall OS measured in colonic mucosa and pain experienced by IBD patients who required surgical interventions due to disease exacerbation and failed conservative treatment. In this study neither pain severity, nor its frequency or influence on motor activity was significantly correlated with OS markers. However, studies on antioxidants, such as propionyl-L-carnitine or camel’s milk show that their use helps diminish abdominal pain in IBD patients and improve their quality of life [8]. Zingiber officinale, a well-known antioxidant also helped increasing the quality of life, decreased disease activity index and OS in patients with UC [40]. On the other hand, diarrhea, one of the most debilitating and painful symptoms in IBD, has been found to be caused by OS. Moreover, the inflammatory response in DSS inflammation model was due to transmural oxidative stress, which in turn along with proinflammatory cytokines led to smooth muscle function impairment, causing diarrhea [41].

A limitation of our study was that it was performed on a relatively small group of IBD patients and the lack of follow-up. Moreover, our control patients were primarily hospitalized due to bowel malignancies. Also, we did not analyze the potential effect of anesthesia and surgery on the OS.

Further studies are warranted to confirm our observations. The major strength was that the study was multifactorial.

## 5. Conclusions

Our study showed that IBD patients who fail to respond to conservative treatment and need surgical intervention present increased lipid peroxidation (measured by TBARS) and decreased level of endogenous antioxidants (SOD and GPX activity and total GSH level). However, subjective pain sensation reported by IBD patients does not correlate with markers of oxidative stress measured in colonic mucosa. Whether the hypothesis of OS influencing pain experienced by IBD patients holds true will require further prospective studies.

## Figures and Tables

**Table 1:** Socio-demographic data of inflammatory bowel disease (IBD) patients and controls.

	Control Group	IBD Patients
Subjects, *n* (%)	32 Sigmoid cancer 11 (34.4%) Ascending colon cancer 4 (12.5%) Hepatic flex. cancer 2 (6.3%) Splenic flex. cancer 1 (3.1%) Caecal cancer 1 (3.1%) Rectal cancer 5 (15.6%) Diverticulosis 2 (6.3%) Sigmoid fistula 1 (3.1%) Ileus 3 (9.4%) Colon reconstruction 2 (6.3%)	31 CD 18 (58.1%) UC 13 (41.9%)
Sex		
Men, *n* (%)	13 (40.6%)	17 (54.8%)
Women, *n* (%)	19 (59.4%)	14 (45.2%)
Age, year	62.28 ± 16.22	38.87 ± 12.64
BMI, kg/m^2^	24.45 ± 4.05	22.20 ± 4.33

Abbreviations: BMI: Body mass index; CD: Crohn’s disease; UC: Ulcerative colitis.

**Table 2:** Clinical data of inflammatory bowel disease (IBD) patients and controls.

	Control Group	IBD Patients
Duration of disease, *n* (%)		
<5 years		17 (54.8%)
5–10 years		5 (16.1%)
10–15 years		3 (9.7%)
>15 years		6 (19.4%)
Number of stools per day		
0–5 a day	26 (81.25%)	20 (64.5%)
5–10 a day	5 (15.6%)	8 (25.8%)
10–15 a day	-	2 (6.5%)
>15 a day	1 (3.1%)	1 (3.2%)
Extraintestinal symptoms, *n* (%)		9 (29.0%)
Use of analgesics, *n* (%)	5 (15.6%)	11 (33.3%)
NSAIDs	3 (9.4%)	7 (22.6%)
Opioids- Tramadol	2 (6.3%)	4 (12.9%)
Biological treatment, IFX, *n* (%)		2 (6.45%)
CDAI, points		305.61 ± 109.87
MAYO, points		2.65 ± 0.74
Vitamin D3 supplementation, *n* (%)		
None	23 (71.9%)	21 (67.7%)
2000 IU	9 (28.1%)	7 (22.6%)
4000 IU		2 (6.5%)
10,000 IU		1 (3.2%)
Total Antioxidant, mM/mg	0.034 ± 0.008	0.032 ± 0.010
SOD activity, U/mg	4.703 ± 1.611	3.125 ± 1.439
CAT activity, U/mg	0.411 ± 0.303	0.755 ± 0.468
Total GSH, µM/mg	0.212 ± 0.104	0.165 ± 0.125
GSSG, µM/mg	0.131 ± 0.082	0.078 ± 0.076
GPX activity, U/mg	1.230 ± 0.742	0.863 ± 0.810
Total COX activity, U/mg	0.105 ± 0.037	0.098 ± 0.037
COX-1, %	34.285 ± 11.278	71.445 ± 12.094
COX-2, %	65.715 ± 11.278	28.555 ± 12.094
TBARS, µM/mg	0.071 ± 0.039	0.184 ± 0.193
CRP, mg/L	2.203 ± 5.948	66.037 ± 64.267

Abbreviations: CAT: Catalase; CDAI: Crohn’s Disease Activity Index; COX: Cyclooxygenase; CRP: C-reactive protein; GPX: Glutathione peroxidase; GSH: Glutathione; GSSG: Oxidized glutathione; IFX: Infliximab; NSAID’s: Non steroid anti-inflammatory drugs; SOD: Superoxide dismutase; TBARS: Thiobarbituric acid reactive substances.

**Table 3:** Laitinen Pain Scale’s results in enrolled IBD patients and controls.

Factor	Subjective Evaluation	Points	Controls *n*, %	IBD *n*, %
Pain intensity	Without pain	0	10 (31.3%)	4 (12.9%)
	Mild	1	10 (31.3%)	10 (32.3%)
	Strong	2	10 (31.3%)	7 (22.6%)
	Very strong	3	2 (6.3%)	6 (19.3%)
	Not sustainable	4	0	4 (12.9%)
Pain frequency	Does not occur	0	13 (40.6%)	5 (16.1%)
	Periodical	1	10 (31.3%)	12 (38.7%)
	Frequent	2	4 (12.5%)	7 (22.6%)
	Very frequent	3	3 (9.4%)	4 (12.9%)
	Continuous	4	2 (6.3%)	3 (9.7%)
Painkillers’ intake	Without medication	0	16 (50.0%)	7 (22.6%)
	Periodically	1	8 (25.0%)	16 (51.6%)
	Permanently—small doses	2	5 (15.6%)	2 (6.5%)
	Permanently—big doses	3	3 (9.4%)	6 (19.4%)
	Permanently—very big doses	4	0	0
Motor activity limitation	None	0	19 (59.4%)	8 (25.8%)
	Partial	1	6 (18.8%)	8 (25.8%)
	Demanding partial help/making work difficult	2	3 (9.4%)	11 (35.5%)
	Demanding partial help/making work impossible	3	4 (12.5%)	2 (6.5%)
	Demanding full help/preventing self sufficiency	4	0	2 (6.5%)

## Data Availability

The data presented in this study are available in article.
